# Immediate effect of acupuncture on pelvic floor muscles function for stress urinary incontinence in women: study protocol for a randomized and sham-controlled trial

**DOI:** 10.3389/fmed.2026.1815206

**Published:** 2026-06-03

**Authors:** Juan-Juan Li, Bing-Li Chen, Xi Wang, Ya-Juan Ren, Xiu-Ling Song, Yue-Lai Chen

**Affiliations:** 1 Acupuncture Department, LongHua Hospital Shanghai University of Traditional Chinese Medicine, Shanghai, China; 2 Acupuncture Department, Shanghai University of Traditional Chinese Medicine Yueyang Hospital of Integrated Traditional Chinese Medicine and Western Medicine, Shanghai, China; 3 Shanghai University of Traditional Chinese Medicine, Shanghai, China

**Keywords:** acupuncture, immediate effect, pelvic floor muscle function, stress urinary incontinence(SUI), study protocol

## Abstract

**Introduction:**

Stress urinary incontinence (SUI) is a common condition in middle-aged and elderly women, significantly impacting their quality of life. Weakening of pelvic floor muscle function is considered one of the main factors contributing to SUI. Despite the established clinical use of acupuncture therapy in treating stress urinary incontinence (SUI), the exact mechanisms underlying its effectiveness require further exploration. This study aimed to assess changes in pelvic floor muscle function during acupuncture using surface electromyography (sEMG). This trial will focus on investigating whether the mechanism by which acupuncture alleviates symptoms in SUI patients is related to the improvement of pelvic floor muscle function.

**Methods:**

This study will be a randomized, single-blind, and sham-controlled trial. The study will recruit 76 female patients diagnosed with SUI. They will be randomly allocated to two groups: acupuncture and sham acupuncture, in a 1:1 ratio. Each group of subjects will receive 10 min of acupuncture treatment at RN3 (Zhongji). Outcomes will be assessed at three time points: baseline, during intervention, and 10 min post-intervention, using pelvic floor muscle sEMG. The primary outcome will be the difference in mean amplitude (μV) of the sEMG recordings during the 10-second tonic contraction phase between the acupuncture and sham acupuncture groups. Secondary outcomes will include assessments of amplitude, variation coefficient, and relaxation time across the other phases of the sEMG evaluation. The trial will record adverse events related to the intervention.

**Discussion:**

This study aims to observe the real-time impact of acupuncture on pelvic floor muscle function using sEMG and to explore how acupuncture exerts its effects.

**Clinical trial registration:**

https://www.chictr.org.cn/, Identifier ChiCTR2200059686.

## Introduction

Stress urinary incontinence (SUI) is a prevalent pelvic floor dysfunction in women (1). Based on the International Continence Society status report, 25%−45% of women occasionally experience urinary incontinence, with SUI accounting for 50% of all cases (2). A study in 2022 showed that (3), over 8.93 million people in China were diagnosed with SUI. SUI occurs in women of different ages, with the highest occurrence in women who have given birth or reached menopause. Various factors such as obesity, estrogen levels, age, and mode of delivery contribute to its development (4). SUI is caused by a variety of clinical triggers, including coughing, sneezing, and strenuous exercise (5). While SUI is not life-threatening, its symptoms can cause inconvenience in daily life, affect social activities, and even potentially lead to psychological disorders, along with a range of social and economic issues (6).

Acupuncture has been shown to relieve symptoms associated with SUI. Randomized controlled trials and meta-analyses have provided evidence supporting the effectiveness of acupuncture in the treatment of SUI, with acupuncture decreasing the frequency and amount of urine leakage (7, 8). Acupuncture treatment has been included as a complementary and alternative therapy in clinical guidelines (1, 9). Despite the proven effectiveness of acupuncture, the potential mechanism underlying its treatment of SUI remains to be elucidated.

One of the etiological factors leading to SUI is the weakening of pelvic floor muscle (PFM) function. The PFM plays an important role in the supporting structure of the pelvic floor. When the supporting structure of the pelvic floor is unstable, overactivity of the bladder neck and proximal urethra may occur. SUI occurs when the abdominal pressure increases and exceeds the urethral closure pressure (10). A previous case report observed the immediate effect of acupuncture on the supporting structure of the pelvic floor (11). However, there is no direct evidence that acupuncture treats SUI by improving the function of PFM, which requires further study.

Clinical methods for assessing PFM function include vaginal palpation, pelvic floor muscle surface electromyography (sEMG), and pelvic floor ultrasound. Vaginal palpation is subjective, and ultrasound only reflects structural changes. In contrast, sEMG provides an objective measure of muscle unit electrical signals, offering a real-time evaluation of PFM functional changes (12).

In light of the above, we hypothesize that acupuncture may improve the patients' PFM function as a potential mechanism for treating SUI. We will use pelvic floor muscle sEMG to evaluate muscle function and compare the immediate effects of acupuncture and sham acupuncture (SA).

## Methods

### Study design

**Figure 1:**
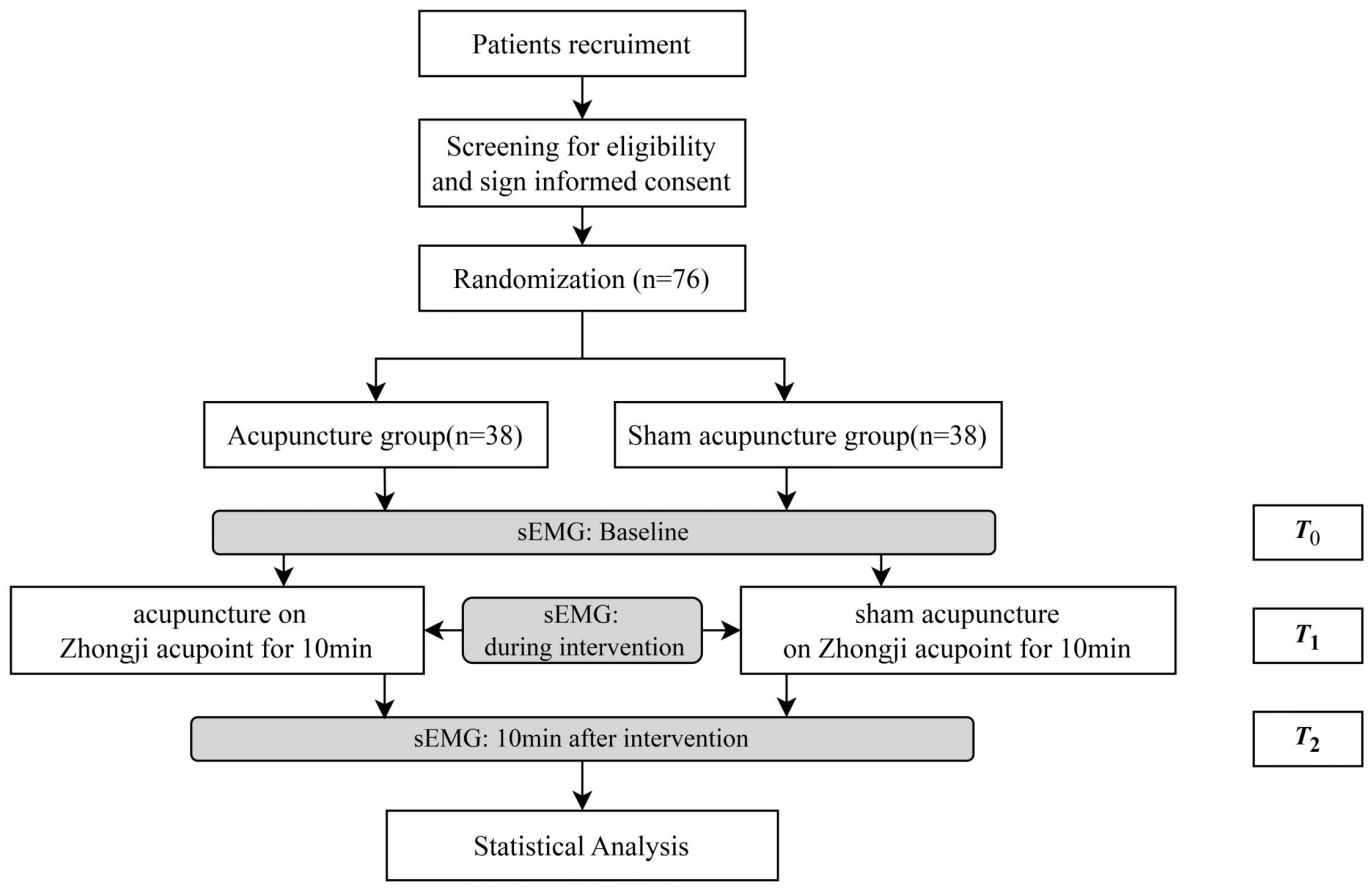
Study flowchart. sEMG, surface electromyography, T0, baseline; T1, during intervention; T2, 10 minutes post-intervention.

**Figure 2:**
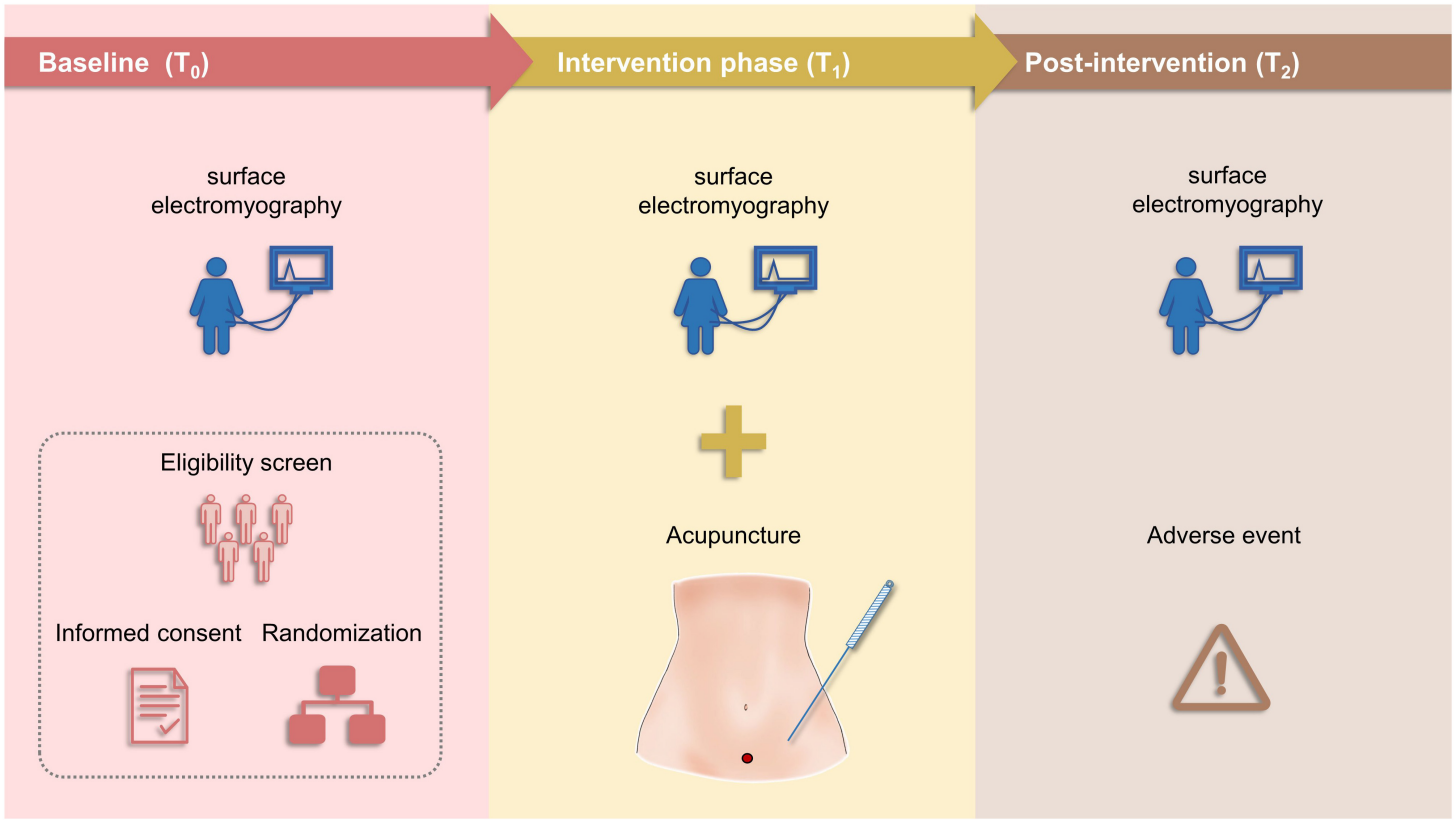
Time schedule T0, baseline; T1, during intervention; T2, 10 minutes post-intervention.

This trial is designed in accordance with the Standard Protocol Items: recommendations for Interventional Trials (SPIRIT) (13). The trial protocol version is V3.0. It has been approved by the Medical Ethics Committee of Longhua Hospital, Shanghai University of Traditional Chinese Medicine (Approval Number: 2022LCSY008) and registered at the Chinese Clinical Trial Registry (ChiCTR2200059686); any changes in the protocol will be reported. The study flowchart is shown in Figure 1, and the time schedule is shown in Figure 2.

### Patient recruitment

This randomized, sham-controlled, single-blind trial will be conducted at Longhua Hospital, Shanghai University of Traditional Chinese Medicine in Shanghai. Potential patients with SUI will be recruited both online and offline, from outpatient clinics by clinicians, and from recruitment posters on hospital websites. After expressing interest in the study, patients will be screened for eligibility by trained staff using the inclusion and exclusion criteria below. A written informed consent form will be obtained from all participants before they are randomized. They will have the right to withdraw at any time, and their personal data will be used exclusively for this study.

### Participants

#### Criteria for diagnosis

The following criteria will be used for the diagnosis of SUI in female patients (14):

1. Involuntary external urethral urinary leakage caused by increased abdominal pressure triggered by sneezing, coughing, laughing, exercise, or other events;
2. 1-h urine pad test indicating weight gain >1 g;
3. Absence of other urinary symptoms, including frequency or urgency.
4. The severity of SUI is determined by the leakage during the 1-hour urine pad test:

(I) ≤ 1 g (normal);
(II) >1 g but <10 g (mild);
(III) ≥10 g but <50 g (moderate);
(IV) ≥50 g (severe).

#### Criteria for inclusion

1. Patients with mild to moderate SUI mentioned in the diagnostic criteria above;
2. Married women between the ages of 40 and 70 years;
3. Participants must enroll in the study voluntarily and sign the informed consent form.

#### Criteria for exclusion

1. History of urinary incontinence surgery;
2. Pelvic organ prolapse ≥ grade II;
3. Residual urine volume > 30 ml;
4. Symptomatic urinary tract infection;
5. Urological deformity;
6. Unable to walk and/or run and/or climb stairs;
7. Acupuncture phobia or metal allergies;
8. Continuing to take medicines (for example, midodrine hydrochloride and duloxetine hydrochloride) that may affect bladder function and/or receiving other urinary incontinence treatment;
9. Serious heart, kidney, liver, brain, or blood system disease, mental disorder, diabetes, medulla spinalis disease, multiple system atrophy, and cauda equina disease;
10. Pregnancy.

### Randomization

This study was designed as a randomized, sham-controlled trial. The randomization sequence was generated by an independent statistician using a computer-generated random number method (SPSS v26.0; IBM Corp., Armonk, NY, USA). Eligible patients were randomly allocated in a 1:1 ratio to either the acupuncture group or the SA group. The allocation sequence was prepared as sequentially numbered cards and sealed in opaque envelopes bearing corresponding identification numbers. After participants were formally enrolled and completed baseline assessments, an independent researcher not involved in the intervention or outcome assessment opened the envelopes sequentially according to the order of enrollment to obtain the allocation information. The group assignment was then promptly communicated to the acupuncturist responsible for delivering the intervention.

Throughout the study, investigators, outcome assessors, and data analysts will be blinded to group allocation. Strict allocation concealment procedures were implemented to minimize potential selection bias. The study design and reporting adhered to the CONSORT guidelines for randomized controlled trials.

### Blinding

Due to the specificity of the intervention, acupuncturists cannot be blinded. The acupuncture needles used in the acupuncture group and the SA group have different lengths, but the length of the needles exposed on the skin is the same to ensure blinding of the sEMG examiners. During the study period, an appointment-based patient consultation system will be implemented, and independent consultation rooms will be provided. Patients participating in the trial will receive the intervention at different time points to avoid mutual communication. Patients will also wear eye masks to ensure blinding. After the intervention, the blinding effect on the patients will be tested using the following method: “Which group do you think you belong to? (A) Acupuncture; (B) SA; (C) Uncertain.” In addition, data entry and statistical analysis personnel will be blinded to the group allocation.

### Interventions

**Figure 3:**
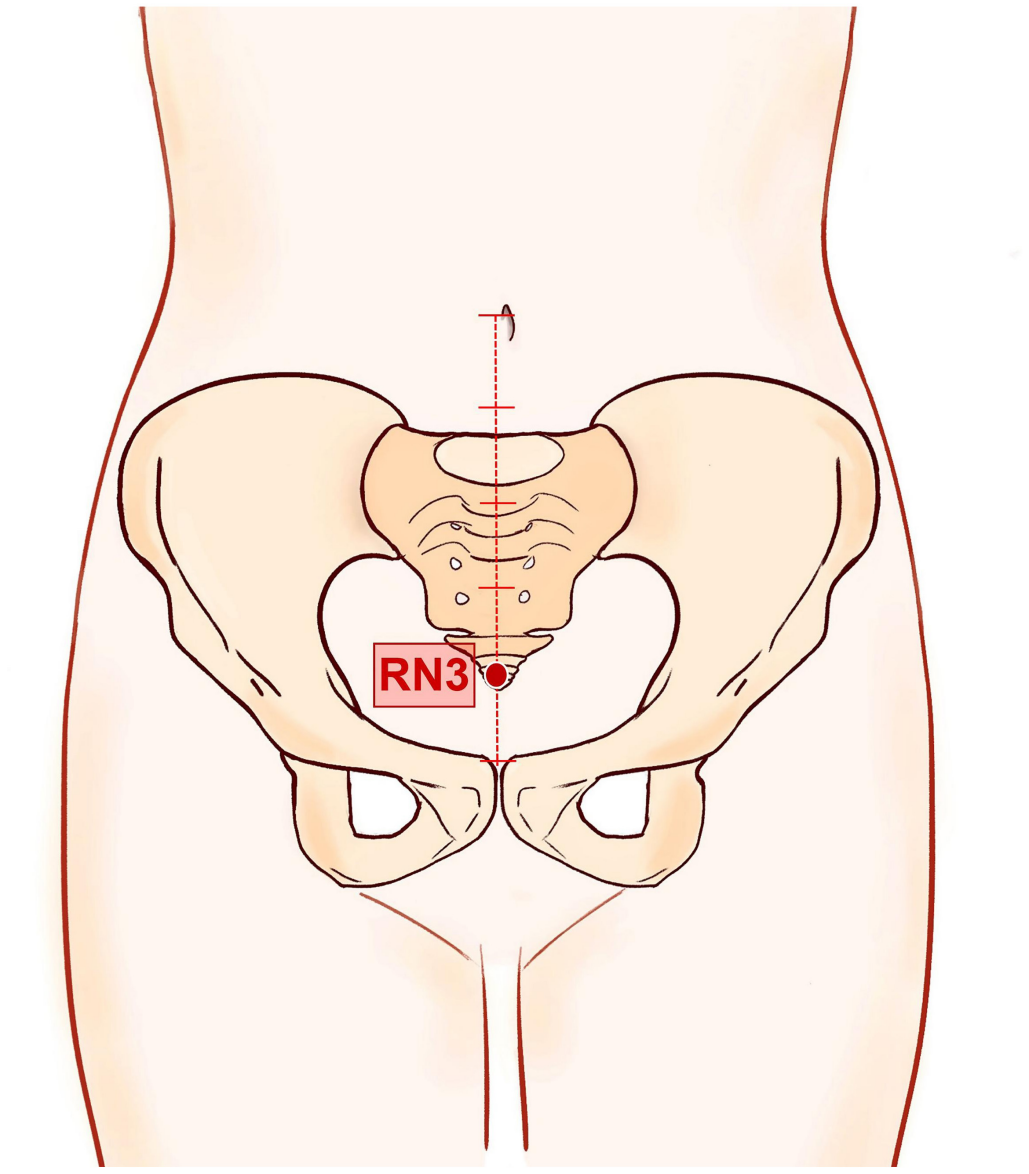
Location of the acupoint. Zhongji (RN3) acupoint placement: fourth of five evenly dispersed intervals from navel to symphysis pubis.

In order to ensure the consistency of interventions, all acupuncture treatments will be carried out exclusively by a licensed and highly experienced acupuncturist (CYL). Before the intervention, the acupuncturist will be trained in the correct use of the SA tool. All participants will receive a single 10 min acupuncture treatment at RN3, which is situated four-fifths of the way down from the navel to the pubic symphysis (Figure 3).

**Figure 4:**
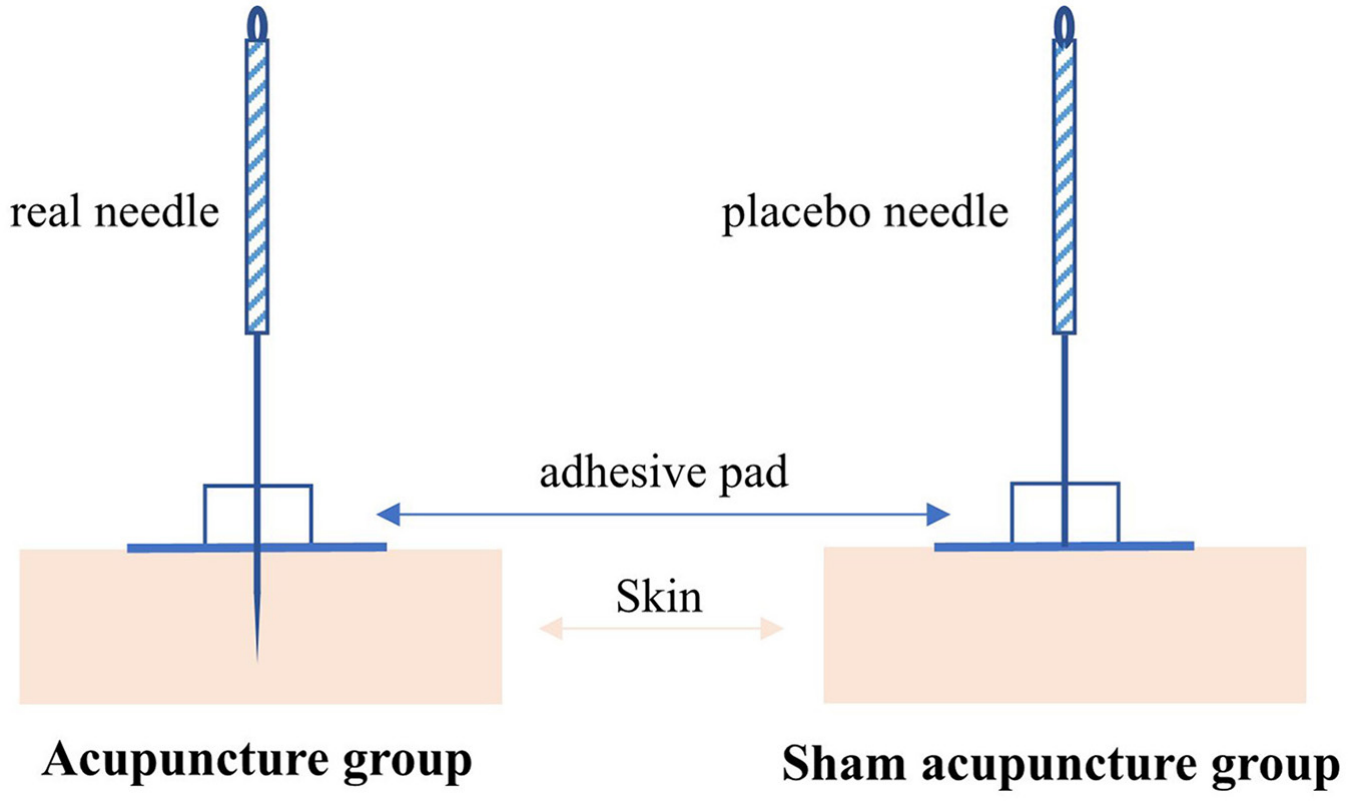
Acupuncture and Sham acupuncture manipulation.

Before the intervention, the acupuncturist will disinfect their hands and RN3 with 75% alcohol. In both groups, a blinding device will be applied to RN3. This device will consist of a sterile, cylindrical polyethylene foam pad (5 mm in thickness, 10 mm in diameter; Suzhou Medical Equipment Factory, China) featuring an adhesive base (Figure 4), designed to maintain the acupuncturist's blindness to the intervention. The feasibility of this blinding device has been previously established (15).

#### Acupuncture group

For the acupuncture group, the real needle (0.30-mm gauge, 75-mm long, Hwato brand, Suzhou Medical Appliance Factory, China) will be used. The needle will be inserted through an adhesive pad into the skin, with a depth of 25–40 mm at RN3. After the needle is inserted, the acupuncturist will perform rapid alternating clockwise and counterclockwise twisting, as well as slight lifting and thrusting, until the patient experiences De qi (characterized by soreness, numbness, heaviness, and distension). The acupuncture treatment will last for 10 min.

#### Sham acupuncture group

For the SA group, a placebo needle with a blunt tip (0.30-mm gauge, 25-mm long, Hwato brand, Suzhou Medical Appliance Factory, China) will pierce the adhesive pad without penetration, merely pressing against it, and will be retained in place for 10 min.

### Outcomes

#### The primary outcome

Mean amplitude during the 10-s tonic contraction phase.

#### The secondary outcomes

(1) The mean amplitude in the prerest phase; (2) The variation coefficient in the prerest phase; (3) The maximum amplitude of five rapid contractions; (4) The relaxation time of five rapid contractions; (5) The variation coefficient in the 10-s tonic contraction phase; (6) The mean amplitude in the 60-s endurance contraction phase; (7) The variation coefficient in the 60-s endurance contraction phase; (8) The mean amplitude in the postrest phase; (9) The variation coefficient in the postrest phase.

Pelvic floor muscle sEMG will be measured using the MLD B4Plus (Biological feedback instrument, Mailande Medical Technology Co., Nanjing). Pelvic floor muscle sEMG will collect muscle movement potentials through a vaginal probe, providing insights into the strength, tone, and coordination of PFM (16, 17). The insertion of the vaginal probe will be conducted by a trained professional, with patients following the device's instructions to complete the assessment. The sEMG evaluation consists of five phases, assessing parameters such as amplitude, relaxation time, and the variation coefficient, which are indicative of clinical significance, as shown in Table 1. This assessment aids clinicians in gaining a comprehensive understanding of patients' PFM function.

**Table 1:** Outcomes of pelvic floor muscle sEMG.

Domain	Meaning	Item	Unit
Outcomes			
sEMG in the prerest phase	measure the muscle tension of the PFMs in the resting status	mean amplitude‡	μV
		variation coefficient‡	
sEMG of five rapid contractions	measure the function of the fast-twitch fibers of the PFMs	maximum amplitude‡	μV
		relaxation time‡	s
sEMG in the 10-s tonic contraction phase	measure the function of the slow-twitch fibers of the PFMs	mean amplitude†	μV
		variation coefficient‡	
sEMG in the 60-s endurance contraction phase	measure the endurance of the slow-twitch fibers of the PFMs	mean amplitude‡	μV
		variation coefficient‡	
sEMG in the postrest phase	measure the recovery function of the PFMs	mean amplitude‡	μV
		variation coefficient‡	

†Primary outcome.

‡Secondary Outcomes.

sEMG, surface electromyography.

The sEMG measurements will be recorded at three time points: baseline (T_0_), during intervention (T_1_), and 10 min post-intervention (T_2_).

### Safety evaluation

The occurrence, duration, and severity of adverse events (AEs), such as fainting, broken needles, unbearable itching at needle insertion sites (VAS ≥ 8), severe post-needling pain > 2 h (VAS ≥ 4), abscess, subcutaneous hematoma, infection, other discomforts (i.e., fatigue, dizziness, or palpitations), and how the event is resolved (or not) will be recorded in a Case Report Form (CRF). Serious adverse events are life-threatening events such as hospitalization or severe incapacity. Serious adverse events will be reported to the Data and Safety Monitoring Board within 24 h for a decision on whether or not to continue tracking.

### Data management and monitoring

#### Data recording

The evaluators responsible for recording the subjects' CRF will undergo standardized training before the experiment to ensure clear and standardized recording methods.

#### Data audit

At the end of each subject's treatment, the researchers will submit the CRF, Informed Consent Form, and pelvic floor sEMG report to the principal researcher of the unit for review and approval within 3 working days.

#### Data entry

To protect the privacy of the subjects, the collected data will be stored on restricted-access computers. The passwords will be set by two independent researchers who are not directly involved in the study. They will be responsible for entering and verifying all information and data into the computer to ensure data accuracy.

#### Data monitoring

The Medical Ethics Committee of the medical institution will regularly review and track the trial data and progress. All data related to this study will be archived and stored by the participating research hospital from the date of study completion.

### Statistical method

#### Sample size

The sample size was calculated using the PASS 15.0 software. The PFM function will be analyzed by a combination of parameters in the sEMG. According to a previous study [10], the contractility of the slow-twitch fibres in PFM function is the main urinary control factor. Therefore, the change in mean amplitude (μV) of the sEMG during the 10-s tonic contraction phase, which represents the contractility of the slow-twitch fibres, was selected for calculation. Based on pilot study data, the mean ± standard deviation (SD) is 4.1 ± 2.7 μV in the acupuncture group and 1.9 ± 1.6 μV in the SA group. 32 cases will be required in each group (α = 0.05, 1–β = 0.90). Taking into account the 15% dropout rate, 38 cases per group were required. Therefore, an optimal sample size of 76 cases will be required.

#### Data analysis

All data will be analyzed using IBM SPSS Statistics (v26.0; IBM Corp, Armonk, NY, USA). The analysis will be conducted according to the intention-to-treat (ITT) principle, including all randomized participants. Safety analyses will be performed on the safety set, which includes all participants who received acupuncture. For both primary and secondary outcomes, missing data will be handled using generalized estimating equations (GEE), which provide valid statistical inference under the assumption of missing at random (MAR) without the need for explicit imputation. If the overall proportion of missing data exceeds 10%, sensitivity analyses will be performed to assess the robustness of the results to the missing data assumption. Missing data for safety outcomes will not be imputed.

Continuous variables will be presented as mean ± standard deviation (SD) if normally distributed, or as median (interquartile range, IQR) otherwise. Categorical variables will be summarized as frequencies and percentages. Baseline demographic and clinical characteristics will be compared using the chi-square test or Fisher's exact test for categorical variables, and independent-samples *t*-tests or Mann-Whitney U tests for continuous variables, as appropriate.

For repeated measurements obtained at three time points (T_0_, T_1_, and T_2_), GEE models will be used as the primary analytical approach to account for within-subject correlations. The models will include group, time, and group-by-time interaction terms, with an appropriate working correlation structure selected based on model fit and data characteristics. The group-by-time interaction will be examined first; if significant, simple effects analyses will be conducted, otherwise main effects will be interpreted. Multiple comparisons will be adjusted using the Bonferroni correction.

All adverse events (AEs) and serious adverse events (SAEs) will be listed. AEs will be classified as acupuncture-related or non-acupuncture-related events. The incidence of AEs will be summarized as the number and percentage of participants in each group and compared using Fisher's exact test. Given the exploratory nature of safety analyses, *P-*value will not be adjusted for multiple comparisons.

Effect sizes along with their 95% confidence intervals (CIs) will be reported. All statistical tests will be two-sided, and a *P* value <0.05 will be considered statistically significant.

## Discussion

Currently, the most recognized pathophysiological mechanisms of SUI are based on pelvic floor anatomy. Both the “hammock hypothesis” proposed by DeLancey (18) and the “Integral Theory” proposed by Petros (19) suggest that impaired PFM function is a key contributing factor in SUI (18, 20). The PFM plays a central role in maintaining continence by providing structural support to the urethra and stabilizing pelvic organs (18, 21, 22). From a functional perspective, continence depends on the coordinated activity of different muscle fiber types within the PFM. Type I (slow-twitch) fibers are primarily responsible for the static support function of the pelvic floor, maintaining continuous urethral support and closure pressure under resting conditions, whereas Type II (fast-twitch) fibers contribute to its dynamic function by being rapidly recruited during increases in intra-abdominal pressure to generate quick and forceful contractions. Disruption of this coordinated mechanism leads to reduced urethral support and insufficient closure pressure, ultimately resulting in urinary leakage (23).

Surface electromyography (sEMG) is an objective and quantitative method for assessing pelvic floor muscle (PFM) function, typically based on the Glazer protocol (24). During muscle contraction, motor units generate bioelectrical signals, and these cellular electrophysiological characteristics are often the first to change when muscle fibers are damaged. By recording these bioelectrical signals via a vaginal probe, sEMG captures the motor potentials during muscle contraction. This enables real-time evaluation of muscle function. Compared with vaginal palpation and manometry, which are either subjective or limited to pressure measurement (25), sEMG offers a multidimensional evaluation of PFM function and has been widely used as an outcome measure in patients with SUI (16). Previous studies have shown that, compared with healthy individuals, women with SUI exhibit reduced muscle contraction and coordination as measured by sEMG (26–28).

In sEMG parameters, the prerest and postrest phases refer to the muscle activity states before and after contraction, respectively. The amplitude in the prerest phase reflects the muscle tension of the PFMs in the resting status; the amplitude in the postrest phase reflects the recovery function of the PFMs. The five rapid contraction phase is used to assess Type II muscle fibers, while the 10-s tonic contraction phase evaluates Type I muscle fibers. The amplitude during this phase reflects the contraction capacity of both Type I and Type II fibers. The 60-s endurance contraction phase primarily reflects the fatigue resistance of Type I muscle fibers. Additionally, variability parameters can reflect the stability of muscle function (29). As the number of activated motor units in the pelvic floor muscles increases, the sEMG amplitude rises, indicating enhanced muscle contraction strength (30). Improvement in pelvic floor muscle function helps stabilize the urethral position and increase urethral closure pressure, thereby alleviating urinary leakage symptoms. In daily life, continence primarily relies on the pelvic floor muscle's ability to provide sustained, tonic support to the urethra, a function predominantly mediated by Type I muscle fibers (31). Therefore, the mean amplitude during the 10-s tonic contraction phase was selected as the primary outcome measure in this study.

As a non-pharmacological therapy, acupuncture is widely used in the treatment of SUI in China. Clinical studies have confirmed the efficacy of acupuncture in the treatment of SUI (8, 32). A large-sample study involving 504 participants has demonstrated that the effects of acupuncture are significantly superior to placebo acupuncture (7). Although acupuncture has been shown to alleviate SUI symptoms, the mechanism behind this intervention remains unclear.

**Figure 5:**
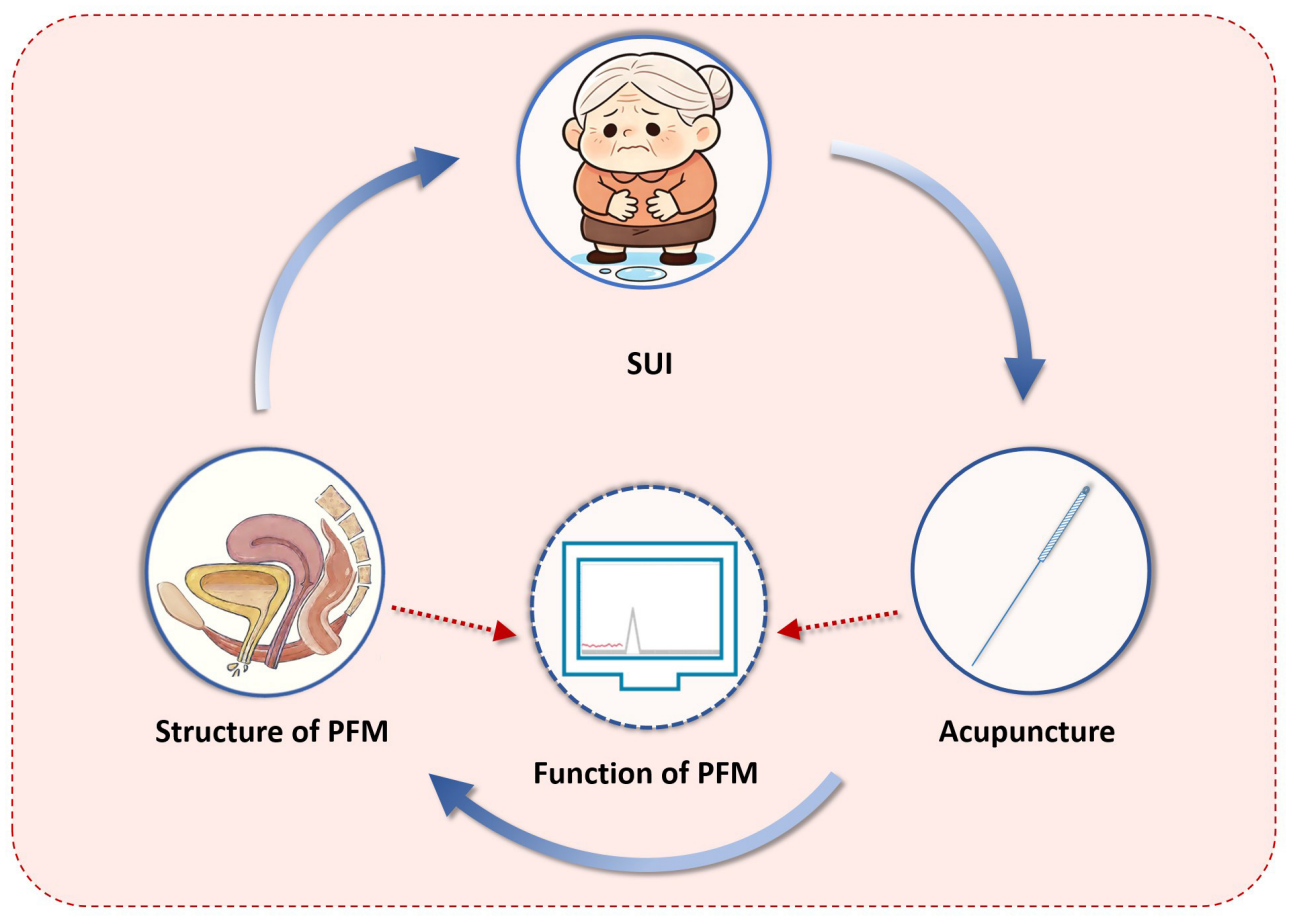
Relationship between SUI, acupuncture, and function of PFM. SUI, stress urinary incontinence; PFM, pelvic floor muscle.

Studies have shown that the pelvic floor structure of SUI patients is altered (33, 34). PFM can maintain the stability of the pelvic floor structure. The role of PFM in supporting pelvic organs and controlling urination is well recognized. Dysfunctional PFM may lead to structural changes in the pelvic floor, which in turn may exacerbate SUI symptoms. In our preliminary study (11), we observed changes in pelvic floor structure in SUI patients following acupuncture at RN3. Based on this finding, we hypothesize that the therapeutic effects of acupuncture may be mediated through the modulation of pelvic floor muscle function. Furthermore, muscle responses can occur rapidly following stimulation, and these early changes may precede observable symptom improvement. A previous study demonstrated that a single acupuncture session can induce changes in muscle strength (35). Therefore, this study adopts a single-intervention design to observe immediate changes in PFM function, providing mechanistic insights into how acupuncture modulates PFM activity at an early stage. This complements future research focusing on long-term clinical outcomes. The use of sEMG in this study allows for a more objective and real-time assessment of acupuncture's effects on PFM function (see Figure 5). As far as we know, this is the first randomized controlled trial to use pelvic floor sEMG to observe functional changes during acupuncture treatment.

In this study, a single acupoint—RN3 was selected to investigate its acupoint-specific effects while minimizing confounding influences from multi-acupoint interventions. Previous studies support the potential role of RN3 in regulating lower urinary tract function. A case report (11) suggested that acupuncture at RN3 may influence pelvic floor-related functional parameters in the short term, and an experimental study (36) has demonstrated that stimulation at RN3 can modulate bladder activity. According to Traditional Chinese Medicine theory, RN3 is the Front-Mu point of the bladder and a key acupoint of the Ren meridian, with functions in regulating bladder activity and lower urinary tract function (37). From a mechanistic perspective, stimulation of RN3, located in the lower abdomen, may activate afferent nerve fibers, thereby modulating sacral reflex pathways involved in continence control. This neuromodulatory effect may facilitate motor unit recruitment and thereby contribute to the modulation of PFM function.

To ensure both physiological effectiveness and methodological rigor, the intervention duration will be standardized to 10 min. This duration aligns with a prior study (38) on the immediate effects of acupuncture and is considered sufficient to induce and maintain the “Deqi” sensation, which is widely regarded as a prerequisite for therapeutic efficacy (39). Moreover, a controlled and relatively short intervention period helps minimize participant discomfort and reduces potential interference with real-time sEMG recordings, thereby enhancing data reliability. In this study, in order to observe the effect of acupuncture on PFM function, three time points will be set: baseline, during intervention, and 10 min post-intervention. The “during intervention” time point will reflect the changes in PFM function when patients receive acupuncture, aiming to explore the mechanism of acupuncture efficacy. Additionally, the “10 min post-intervention” time point will also include a PFM function assessment to observe the sustained effect of acupuncture on PFM function.

To enhance the feasibility of the blinded method, a non-penetrating sham needle design combining a blunt-tipped needle with a foam pad will be applied. The blunt tip will prevent skin penetration while providing tactile stimulation to the epidermis, thereby minimizing the “limbic touch response” (40) induced by superficial needling and offering an effective masking effect. The operator will remove the foam pad upon needle withdrawal to verify that the skin remains intact. Non-penetrating sham acupuncture has been shown to be particularly suitable for evaluating immediate physiological effects and may improve control reliability, particularly in participants with prior acupuncture experience (41). Acupuncture can influence muscle strength (42); thus, we used a non-penetrating sham needle design to avoid affecting muscle tension or strength. The feasibility and scientific validity of this sham acupuncture approach have been previously verified (15, 41). During the study period, an appointment-based consultation system and independent consultation rooms will be used to prevent inter-patient communication. Pelvic floor muscle sEMG assessments will be performed at different time points to ensure objective and reliable outcome assessment.

Nevertheless, the design of this study has several limitations that should be acknowledged. First, we will assess only the immediate effects of a single 10 min intervention, without evaluating long-term efficacy. Although this design will capture early functional responses of the pelvic floor and provide mechanistic insights, it will remain unclear whether these changes translate into sustained improvements in muscle function. In addition, functional or patient-reported outcomes for SUI (e.g., International Consultation on Incontinence Questionnaire-Urinary Incontinence Short Form score and 1-h urine pad test) will not be included in the present study, because the assessment periods required for these measures could not be aligned with the real-time sEMG measurement time points (baseline, during intervention, and 10 min post-intervention). Future studies are warranted to further explore the optimal treatment course and long-term efficacy of acupuncture.

Second, although the single-acupoint design will enhance mechanistic interpretability, it may not fully reflect routine clinical practice, in which multi-acupoint protocols are commonly used. Previous studies suggest that different mechanisms may exist between single and multi-acupoint acupuncture (43), which can be explored in subsequent studies. Moreover, the tactile stimulation produced by the blunt-tipped needle, along with participants' expectations and preconceived beliefs, may still induce physiological or psychological responses. Although placebo effects are generally more pronounced in subjective outcomes, their influence on objective measures cannot be completely ruled out. At the same time, due to the nature of acupuncture, the acupuncturist will not be blinded. In addition, as this study is conducted in a single clinical center with a specific patient population, the generalizability of the findings to other settings and populations may be limited.

## Conclusion

The outcomes are expected to show the changes in PFM function during acupuncture to explore the mechanism of acupuncture. Meanwhile, Type I and Type II muscle fibers in PFM will be subdivided. This trial is the first to use pelvic floor muscle sEMG to explore the immediate effect of acupuncture on PFM function. confidentiality of their data.
